# Supplementary material for: Breast tenderness and swelling experiences related to menstrual cycles and ovulation in healthy premenopausal women: Secondary analysis of the 1-year “Prospective Ovulation Cohort”
Source: PLoS One. 2025 May 12;20(5):e0321205. doi: 10.1371/journal.pone.0321205 (PMC12068584; doi:10.1371/journal.pone.0321205)
Supplement: Attachment — Submitted filename: PEER REVIEW COMMENTS.pdf [file pone.0321205.s001.pdf]

TITLE: Breast experiences related to Menstrual cycle and ovulation in healthy premenopausal women: the 1 year "Prospective Ovulation Cohort" Mary wood

COMMENTS

GENERAL COMMENTS

1. Cohort study done using previous secondary data is called a retrospective cohort study or historical cohort study. Secondary data cannot be utilized to carry out a prospective cohort study. ABSTRACT
2. Title should contain "A secondary data analysis "
3. A standard definition of the population used which falls into the pre- menopausal age group bracket. Your population was 20-40 years are they pre-menopausal? Insert your reference.
4. Title is vague should have indicated the specific breast experiences which will be the dependent variable or outcome. As prospective cohort studies must have a timeline for follow up, which indicates the time to develop the outcome (and exemplified as per person per year) not any arbitrary time frame. ABSTRACT
5. For range in the statement (Line 26), the comma should be changed to hyphen "the median breast tenderness was 1.4 (on a 0-4 scale, range 0.0, 3.0)".
6. For CI in the statement (line 27) " in cycles with a mean length of 28.1 days (95% CI 27.5, 28.8)". The comma should be changed to hyphen.
7. Explain how the within woman finding of no statistical significance in breast tenderness impacts on your primary objective of your study and its conclusion. Your conclusion in the abstract should be in line with the objectives, this enables relay a snapshot summary.
8. The study design utilized is limited when temporality needs to be established hence conflicting with your statement in line 52-54 which states " Both women and clinicians need to know what is expected or 'normal' for menstrual cycle related breast swelling and tenderness. We also need to know if breast changes across a cycle reflect ovulatory status", cannot be investigated extensively with regard to casualty.
   INTRODUCTION A.This statement should be referenced " However, most menstrual studies do not assess ovulatory status or use non-valid methods". B. The normal menstrual cycle length is 21-35 days. Reference this statement "To enrol, women needed two consecutive normal-length (21-36 day"
   C. Line 6. How did you account for differential pain perception- minimal pain documented by a respondent may be maximal to respondent (confounding/ propensity scoring)
   D. Line 108: Delete this () from the range and insert a comma, in the statement "as mean (95% confidence interval) or median (range) across two groups". It is a distinct statistical term, a measure of dispersion not a measure of central tendency. E. Line 110: correct analysis in the statement "Based on data distribution (normally vs. non-normally distributed), statistical analyses" analysis is singular while analyses is plural. G. Line 112-113: You used Man Whitney U test {which is utilized for non- parametric data }to compare the mean(a parametric data). H.Line 115-118: What objective are you analyzing using Wilcoxon Signed Rank Test, when you compared the constructed composite "breast tenderness score" with the other parameters in both groups? I. Line 122-123: What objective did breast tenderness and size correlations answer. Always do the statistical analysis which are pertinent to your study objectives. ETHICAL APPROVAL: A.Line 133-134: This statement is not necessary "These data were de-identified so that the

identities of the participants were not known to the researchers analyzing the data". B. This statement should be under the section on ethical approval " They were not financially compensated for study participation". C.Insert the appropriate flow diagram for the study. RESULTS: Documentation should chronologically reflect your objectives to reduce ambiguity. A. Line 167... Table 1: The cut off 1.4 used to dileanate mild tenderness from moderate tenderness should be noted and referenced in your methodology. B. This statement should be re- written "In the 53 women in the Prospective Ovulation Cohort, breast experiences related to the menstrual cycle were reported for 720 cycles, 97% of which were of normal lengths (21-36 days). They were also studied in the 694 cycles with documentable ovulatory status" B. Line 220-224: Indicate if there was a positive correlation noted. Include the figure 3. C. For all your tables the narrative should be below the tables, highlighting important information. DISCUSSION. A. Line 227: This statement contradicts your result which stated that some of the cycle analyzed were in the menstrual phase." breast swelling increases in the days before flow across menstrual cycles in 53 healthy". B. Line 241-244: A causality analysis between estradiol level and breast tenderness / size changes was not done, hence this statement cannot be deduced " Although progesterone levels were 84% lower, estradiol was decreased by only 20% [22]. Based on the results of our study, it is apparent that even this minimal decrease in estradiol was sufficient to result in a perceivable decrease in breast tenderness and swelling". C. Line 247-249: This statement should be under justification not discussion "Although progesterone levels were 84% lower, estradiol was decreased by only 20% [22]. Based on the results of our study, it is apparent that even this minimal decrease in estradiol was sufficient to result in a perceivable decrease in breast tenderness and swelling". D. The objectives of your study didn't include this, hence this recommendation cannot be made. Line 296-297 "The MCD© version used to track breast experiences for our POC did not delineate the specific site or type of breast tenderness – front vs. high-lateral or axillary. Breast tenderness location has been linked to different hormone levels: front or sub-areolar with higher estrogen, and axillary or high lateral with luteal levels of progesterone. Therefore, taking into account breast tenderness locale when comparing experiences of normally ovulatory cycles to SOD may be important in future research" LIMITATIONS. A. Always state the attempts made at circumventing the limitations. B. Limitations due to missing data and occurrence of type 2 error. STRENGTHS B. Line 304 -305: Comparison with shorter durations of previous studies is not the strength of a prospective study. From review of literature (conceptualization phase) using appropriate time of exposure to occurrence of outcome is what gives prospective studies a higher scientific evidence level. B. QBT does not prove ovulation, is implies it likely occurred. Line 305-307 "strength is that all women were QBT©-proven initially normally cycling and ovulatory". C. Include the limitation imposed by use of body basal temperature analysis to extrapolate the duration of luteal phase. D. Include definitions of terms and state reasons why two consecutive cycles were chosen instead of the standard (three). Include appropriate references. F. Include the rational for use of age group 20-41 years. REFERENCES A. Line 358-359 : Remove the abbreviation of the journal , since you wrote the full name. B. References 10, 11 and 17- complete the journal name,

GENERAL SUMMARY

-Confirmation of ovulation occurs post ovulation which is controversial.

1. The statement made on line 52, 53 and 54 can only be hypothesized by an autologous study with specific criteria met thereafter, using e.g the Bradford hill's criteria. A retrospective cohort study cannot determine temporality.
2. The statement made on line 53, is not justified by your study design. To know if breast changes across cycle reflect ovulation status a comparative analytic study should be done.
3. Write a sentence or two: how good physical health and emotional health were quantified.
4. What was your sampling technique? Probability or non- probability (consecutive sampling). These impacts the generalizability of your findings and nuances the conclusion.
5. The multiple linear regression analysis should not be used for categorical variables. For outcome that is categorical so a multivariate logistic regression model should be utilized.
6. State the rational for combining two statistical analytic methods for this study. SPSS or R version can carry out the analysis as a stand alone.
7. Use standard tables and label them appropriately. Write important notes below the table.
