# Supplementary material for: Breast tenderness and swelling experiences related to menstrual cycles and ovulation in healthy premenopausal women: Secondary analysis of the 1-year “Prospective Ovulation Cohort”
Source: PLoS One. 2025 May 12;20(5):e0321205. doi: 10.1371/journal.pone.0321205 (PMC12068584; doi:10.1371/journal.pone.0321205)
Supplement: Attachment — Submitted filename: POC breast-Responses to Editor and Reviewers Feb 11 - Final.pdf [file pone.0321205.s003.pdf]

THE UNIVERSITY OF BRITISH COLUMBIA

Department of Medicine
Division of Endocrinology and Metabolism
Centre for Menstrual Cycle and Ovulation Research
CeMCOR, ***www.cemcor.ubc.ca***
Gordon and Leslie Diamond Health Care Centre
2775 Laurel Street/ Room 4111
Vancouver, B.C. V5Z 1M9
Jerilynn C. Prior MD, FRCPC, Professor
Phone: 604-875-5927
Fax: 604-875-5915

February 11, 2025

Dear Dr. Patrick Ifeanyi Okonta,

When submitting your revision, we need you to address these additional requirements.

1. Please ensure that your manuscript meets PLOS ONE's style requirements, including those for file naming. The PLOS ONE style templates can be found at https://journals.plos.org/plosone/s/file?id=wjVg/PLOSOne_formatting_sample_main_body.pdf and https://journals.plos.org/plosone/s/file?id=ba62/PLOSOne_formatting_sample_title_authors_affiliations.pdf<https://track.editorialmanager.com/CL0/https:%2F%2Fjournals.plos.org%2Fplosone%2Fs%2Ffile%3Fid=ba62%2FPLOSOne_formatting_sample_title_authors_affiliations.pdf/1/010f0194149fe789-71bb2081-c775-402b-af26-b5b01e31d061-000000/Q8tPGFu9EXaoIM_gHIxp6uE6Q9jJ-zPEhRCjHu34xnA=190>.

We have followed these instructions and believe that this manuscript meets PLOS ONE's style requirements.

2. We note that you have indicated that there are restrictions to data sharing for this study. For studies involving human research participant data or other sensitive data, we encourage authors to share de-identified or anonymized data. However, when data cannot be publicly shared for ethical reasons, we allow authors to make their data sets available upon request. For information on unacceptable data access restrictions, please see http://journals.plos.org/plosone/s/data-availability#loc-unacceptable-data-access-restrictions.
Before we proceed with your manuscript, please address the following prompts:

a) If there are ethical or legal restrictions on sharing a de-identified data set, please explain them in detail (e.g., data contain potentially identifying or sensitive patient information, data are owned by a third-party organization, etc.) and who has imposed them (e.g., a Research Ethics Committee or Institutional Review Board, etc.). Please also provide contact information for a data access committee, ethics committee, or other institutional body to which data requests may be sent.

We fully support PLOS ONE's efforts to make scientific primary data more easily available and potentially subject to re-analysis.
However, at the time of recruitment in the mid-1980s, the informed consent document included the scientific analysis and later use of these data. But it did not include that the primary data would be shared. This is a major ethical limitation.

b) If there are no restrictions, please upload the minimal anonymized data set necessary to replicate your study findings to a stable, public repository and provide us with the relevant URLs, DOIs, or

accession numbers. Please see http://www.bmj.com/content/340/bmj.c181.long for guidelines on how to de-identify and prepare clinical data for publication. For a list of recommended repositories, please see https://journals.plos.org/plosone/s/recommended-repositories. You also have the option of uploading the data as Supporting Information files, but we would recommend depositing data directly to a data repository if possible.

Please update your Data Availability statement in the submission form accordingly.

2. In this instance it seems there may be acceptable restrictions in place that prevent the public sharing of your minimal data. However, in line with our goal of ensuring long-term data availability to all interested researchers, PLOS' Data Policy states that authors cannot be the sole named individuals responsible for ensuring data access (http://journals.plos.org/plosone/s/data-availability#loc-acceptable-data-sharing-methods).

We agree that institutional management is more reliable. As the senior author, I am now almost 82 years old!
We are in the process of depositing our de-identified data for this analysis into a University of British Columbia "dataverse" instrument (Borealis) that will be accessible, if appropriate, through non-author university employees.

Additional Editor Comments (if provided):

We respectfully request that the revision of this manuscript proceed without interruption while these data availability issues are being resolved.

Thank you for your consideration of this request.

Detailed responses to reviewers including identification of manuscript changes

Reviewer 1

The authors has looked into an aspect of medicine that has been assumed, their study is therefore opening new grounds for other researchers to look into. But 40 years before the data was analyzed may be a drawback, I recommend a recent study can be done to evaluate the same breast changes.
The statistical analysis is appropriate. The language was standard English and written in an intelligible fashion. The conclusion is supported by the data.

Thank you for your positive assessments of the statistics, language and conclusion.

Unfortunately, at this time funding is not available to collect new data and repeat the initial study which included MCD© and QBT© data over the course of a year for 53 women. We do not believe that is necessary. There is no reason to think that contemporary data collection with repeated analysis would alter our findings. It is unlikely that spontaneously recorded breast tenderness and swelling experiences in relation to ovulatory status would have changed over a 40-year period. When looked at that way, we trust you will agree.

Reviewer 2

1.Cohort study done using previous secondary data is called a retrospective cohort study or historical cohort study. Secondary data cannot be utilized to carry out a prospective cohort study.

Thank you for your careful and comprehensive review.

You are quite correct that this is a secondary analysis. However, it is an analysis of prospectively collected data and thus still considered to have a prospective cohort design. The Menstrual Cycle Diary© (MCD©) data were collected prospectively during the initial study (Prior, 1990, *New England J Med*). In that investigation we used the MCD© for cycle length timing and as a form for recording first morning temperatures used in the Quantitative Basal Temperature© [QBT©] analysis.
However, most MCD data items were not reported until we determined the protocol for secondary analysis of these diary data in the Prospective Ovulation Cohort study analyzing and reporting the MCD©-recorded experiences of the 53 women with at least 8 consecutive cycles (mean 13.6). That published open-access protocol is accessible here http://hdl.handle.net/2429/80682.
This is a prospective study because the participants were followed prospectively (over time into the future) and as stated by Klebanoff & Snowden (2018). It is the timing of data collection with respect to when the follow up/observed outcome takes place that makes a study prospective vs. retrospective, not the timing of the analysis, nor the year when the data were collected.

Klebanoff MA, Snowden JM. Historical (retrospective) cohort studies and other epidemiologic study designs in perinatal research. American Journal of Obstetrics & Gynecology. 2018;219(5):447-50.

ABSTRACT

2. Title should contain "A secondary data analysis "

Thank you for that suggestion. We have added it (Line 4).

3. A standard definition of the population used which falls into the pre- menopausal age group bracket. Your population was 20-40 years are they pre-menopausal? Insert your reference.

The participants included in this study were deemed premenopausal. They had predictable normal length (21-36 day) menstrual cycles with two consecutive normally ovulatory cycles prior to study enrolment. Normal ovulatory status was proven with QBT© determined luteal phase lengths of 10 or more days. Based on the STRAW+10 criteria (Harlow et al., 2012), they would only be considered perimenopausal if they had persistent variability in cycle lengths by 7 days or more in consecutive cycles.

Harlow SD, Gass M, Hall JE, Lobo R, Maki P, Rebar RW, et al. Executive summary of the Stages of Reproductive Aging Workshop + 10: addressing the unfinished agenda of staging reproductive aging. Menopause. 2012;19(4):387-95.

4. Title is vague should have indicated the specific breast experiences which will be the dependent variable or outcome. As prospective cohort studies must have a timeline for follow

up, which indicates the time to develop the outcome (and exemplified as per person per year) not any arbitrary time frame.

Thank you for that suggestion. We have added “breast tenderness and swelling” to the title (Line 3).

5. For range in the statement (Line 26), the comma should be changed to hyphen “the median breast tenderness was 1.4 (on a 0-4 scale, range 0.0, 3.0)”.

We generally prefer a comma between ranges to ensure that a hyphen is not interpreted as a negative number. However, we have accepted your suggestion. See (Line 28)

6. For CI in the statement (line 27) “ in cycles with a mean length of 28.1 days (95% CI 27.5, 28.8)”. The comma should be changed to hyphen.

We have again accepted your suggestion. See (Line 29)

7. Explain how the within woman finding of no statistical significance in breast tenderness impacts on your primary objective of your study and its conclusion. Your conclusion in the abstract should be in line with the objectives, this enables relay a snapshot summary.

We respectfully disagree with the suggestion to describe the primary outcome first.

Given that there is reason to suspect more accuracy of a self-reported experience within- than between women, that the within-woman outcome was our primary objective, and that our within-woman and between women results differed, **we believe that our within-woman findings are important to report in the abstract.** We had only 18 anovulatory cycles in this database given that all women were initially normally cycling and ovulatory. These results highlight the need for future research with more anovulatory cycles for direct comparison to further evaluate our primary objective.

8. The study design utilized is limited when temporality needs to be established hence conflicting with your statement in line 52-54 which states “Both women and clinicians need to know what is expected or ‘normal’ for menstrual cycle related breast swelling and tenderness. We also need to know if breast changes across a cycle reflect ovulatory status”, cannot be investigated extensively with regard to casualty.

You are right that we cannot assess causality. We are only attempting to *describe* breast tenderness and swelling experiences as well as *when within the menstrual cycle* the breast experience changes occurred (temporality).
Our study prospectively followed 53 previously healthy and normally cycling women with self-reported breast experiences (tenderness and size changes) over at least 8 consecutive cycles (mean 13). We were able to show the general trends and characterization of breast changes experienced across cycles that were proven to be ovulatory based on QBT© data, as well as the relationship and cycle phase timing of breast tenderness and swelling throughout all ovulatory cycles.
Given the extensive screening, and the healthy nature of the participants, these findings can be extrapolated to similar populations as a reflection of expected menstrual cycle experience changes.

INTRODUCTION

A. This statement should be referenced "However, most menstrual studies do not assess ovulatory status or use non-valid methods".

We agree that this statement should be referenced. We have added two references, (Lines 51-52)

B. The normal menstrual cycle length is 21-35 days. Reference this statement "To enrol, women needed two consecutive normal-length (21-36 day)

You are right to call this out.

In the late 1970s when planning was underway for data collection, the upper length of the normal menstrual cycle was considered 36 by experts like Dr. Abraham. We've referenced a review of his (Line 73).

C. Line 6. How did you account for differential pain perception- minimal pain documented by a respondent may be maximal to respondent (confounding/ propensity scoring)

Pain scales are entirely subjective, and as you correctly identified, respondents have different pain thresholds and tolerance levels. We were assessing breast tenderness that we expected to be and was usually mild (as the median was 1.4 on a 0-4 scale shows).

We simply scored breast tenderness with the same scale as we used for other things like frustration or sleep problems. Our MCD© data were to evaluate trends and variations in reported experiences throughout an individual's cycle to assess patterns with regards to ovulatory status.

D. Line 108: Delete this ( ) from the range and insert a comma, in the statement "as mean (95% confidence interval) or median (range) across two groups". It is a distinct statistical term, a measure of dispersion not a measure of central tendency.

We have followed your suggestion—see (Lines 112-113)

E. Line 110: correct analysis in the statement "Based on data distribution (normally vs. non-normally distributed), statistical analyses" analysis is singular while analyses is plural.

Thank you. We have changed 'analyses' to 'analysis' (Line 114)

G. Line 112-113: You used Man Whitney U test {which is utilized for non- parametric data }to compare the mean (a parametric data).

In summarizing, I guess our statistical analysis plan became unclear.

We have indicated that we used a non-parametric test for data (such as the ordinal and not interval data in which breast tenderness was scored) that were not normally distributed.

H. Line 115-118: What objective are you analyzing using Wilcoxon Signed Rank Test, when you compared the constructed composite "breast tenderness score" with the other parameters in both groups?

Thank you for this question. In **Table 3** we should have made it clear that our non-parametric but paired analysis of breast experience parameters used this test for within-woman analysis. We've added a footnote to **Table 3** to indicate the use of the Wilcoxon Signed Rank Test for these data.

I. Line 122-123: What objective did breast tenderness and size correlations answer. Always do the statistical analysis which are pertinent to your study objectives.

Thank you for the important reminder to make sure that data analysis responds to stated study objectives.

One of our secondary objectives was to *characterize the timing and variation of normal breast size changes associated with the menstrual cycle* (Lines 301-302). As a component of this analysis, we examined the timing and pattern of both breast tenderness and change in size across a normally ovulatory cycle. These two breast experiences were found to be positively correlated, both increasing towards the end of the luteal phase prior to the onset of menses.

Knowing that increased breast tenderness and increased breast size or swelling, which can contribute to perceived breast tenderness, is a normal premenstrual experience across an ovulatory cycle is valuable information to both clinicians and premenopausal women in the context of menstrual health evaluation.

ETHICAL APPROVAL

A. Line 133-134: This statement is not necessary "These data were de-identified so that the identities of the participants were not known to the researchers analyzing the data".

We have taken this statement out (see Line 142) but referred to de-identified data elsewhere as our ethics confidentiality guideline require.

B. This statement should be under the section on ethical approval " They were not financially compensated for study participation".

We have moved the place in the document in which this is mentioned, as you requested. (Lines 140-142)

C. Insert the appropriate flow diagram for the study.

We **have** inserted the Flow Diagram early in the results to orient the reader to what data we are analyzing (see Lines 168-171)

RESULTS

Documentation should chronologically reflect your objectives to reduce ambiguity.

Thank you for your perspective.

Before the objectives can be understood and data presented, we believe it is important to first introduce the participants in a trial, to show the flow of participants and data through the study, to

show the distribution of the major factor of interest (breast tenderness) and to first provide complete baseline data stratified by the median breast tenderness.

We then show the primary parameters for breast tenderness and size changes for all women, examining whether or not they differ by normally ovulatory status or not.

We are then ready to present our primary analysis of within-woman changes by ovulatory characteristics.

This is the order of the data presentation:

-Fig 1. Flow diagram

- Fig 2. Frequency Distribution of Mean Breast Tenderness

- Table 1. Baseline demographics for all 53 POC Women.

- Table 2. Breast Tenderness and Size Change Parameters for all 53 POC Women.

- Table 3. Within-woman Comparison of Breast Tenderness and Breast Size Changes

- Fig 3. Temporal Pattern of Mean Breast Tenderness and Breast Size Change Related to the Day of Ovulation.

A. Line 167... Table 1: The cut off 1.4 used to dileanate mild tenderness from moderate tenderness should be noted and referenced in your methodology.

We think you must have missed that we presented continuous data on breast tenderness distribution in **Figure 2** earlier. See (Lines 179-182)

B. This statement should be re- written "In the 53 women in the Prospective Ovulation Cohort, breast experiences related to the menstrual cycle were reported for 720 cycles, 97% of which were of normal lengths (21-36 days). They were also studied in the 694 cycles with documentable ovulatory status."

Thank you. We have done as you asked, only splitting it into two sentences. (Lines 154-157)

C. Line 220-224: Indicate if there was a positive correlation noted. Include the figure 3.

We ***have*** indicated the positive correlation ((Line 242)

D. For all your tables the narrative should be below the tables, highlighting important information.

Our strong preference is to introduce the material in the Table before it appears in the document. This seems to us to be a matter of preference; we don't believe it is relevant to the science. With respect, we have thus not followed your suggestion.

DISCUSSION

A. Line 227: This statement contradicts your result which stated that some of the cycle analyzed were in the menstrual phase." breast swelling increases in the days before flow across menstrual cycles in 53 healthy".

Thank you for that observation. However, it is not correct.

We have documented and discussed the follicular and luteal phases, but not the "menstrual phase" anywhere in this document.

B. Line 241-244: A causality analysis between estradiol level and breast tenderness / size changes was not done, hence this statement cannot be deduced "Although progesterone levels were 84% lower, estradiol was decreased by only 20% [22]. Based on the results of our study, it is apparent that even this minimal decrease in estradiol was sufficient to result in a perceivable decrease in breast tenderness and swelling".

These lines simply refer to data from our previously published population-based study of the point prevalence of ovulation in a Norwegian county. We are making inferences as it is appropriate to do in the discussion. You are right that we cannot say assess causality.

C. Line 247-249: This statement should be under justification not discussion "Although progesterone levels were 84% lower, estradiol was decreased by only 20% [22]. Based on the results of our study, it is apparent that even this minimal decrease in estradiol was sufficient to result in a perceivable decrease in breast tenderness and swelling".

Were simply using data we previously published on normally ovulatory versus SOD cycles, data in which we have estradiol and progesterone levels, which we do not have in the current dataset, to try to understand the results that we found in the current study. We have modified the statement to "it is possible that even this minimal decrease in estradiol. . . ." (Lines 270-271)

D. The objectives of your study didn't include this, hence this recommendation cannot be made. Line 296-297 "The MCD© version used to track breast experiences for our POC did not delineate the specific site or type of breast tenderness – front vs. high-lateral or axillary. Breast tenderness location has been linked to different hormone levels: front or sub-areolar with higher estrogen, and axillary or high lateral with luteal levels of progesterone. Therefore, taking into account breast tenderness locale when comparing experiences of normally ovulatory cycles to SOD may be important in future research"

Thank you for that suggestion. We have removed this paragraph.

LIMITATIONS

A. Always state the attempts made at circumventing the limitations.

You are quite correct.

We have mentioned that these are secondary analyses of previously collected data. There was no way we could mitigate this limitation.

B Limitations due to missing data and occurrence of type 2 error.

Good suggestion. We have very few missing data—although we could not assess our outcomes related to ovulation in the 26 cycles (3.6%) that had QBT data that were missing or, for fever at midcycle, could not be analyzed.

Our apparently negative data in Table 3 are likely because we did not have sufficient anovulatory cycles. However, anovulatory cycles are naturally rare in healthy women, and the 18 we observed out of 694 cycles match this expectation. While a Type II error is possible, it is expected due to the biological rarity of anovulation and is a known limitation.
We appreciate this careful review (See Lines 297-298).

STRENGTHS

Line 390305: Comparison with shorter durations of previous studies is not the strength of a prospective study. From review of literature (conceptualization phase) using appropriate time of exposure to occurrence of outcome is what gives prospective studies a higher scientific evidence level.

You are quite correct that it would have been good to know what duration and levels of estradiol and progesterone in the luteal phases of normally ovulatory or short luteal phase cycles would cause breast tenderness or size changes. **We could not know that *a priori.***

We followed accepted scientific processes in performing a thorough literature review prior to the start of this study to analyze what previous research had already been completed on 'normal' menstrual cycle experiences. We found that most of the existing studies were limited by either a lack of documented ovulatory status, or failure to analyze more than 1-2 cycles which only provide a snapshot of an individual's ovulatory health and menstrual experiences.

The duration (at least 8 consecutive cycles reported per woman with mean 13.6) of our study is a strength, separate from the prospective design; It increases the validity, as well as the power of our study which increases with increased sample sizes. Subsequent confirmation of normally ovulatory cycles (beyond two consecutive cycles prior to enrolment) also highlights the health of the 53 POC women who were included in the study.

B. QBT does not prove ovulation, is implies it likely occurred. Line 305-307 "strength is that all women were QBT©-proven initially normally cycling and ovulatory".

You are correct that this language exceeds our certainty. We have changed it to "Another strength is that all women in two consecutive cycles were initially normally cycling and ovulatory based on QBT© prior to enrollment. (Lines 331-333).

C. Include the limitation imposed by use of body basal temperature analysis to extrapolate the duration of luteal phase.

We have shown that QBT© does not carry the interobserver errors of BBT. We do not believe that its use is a limitation.

The gold standard for confirming ovulatory status is a daily mid-cycle transvaginal ultrasound showing collapse of a dominant follicle. These data were collected before that was the gold standard or technically possible.

We have iteratively attempted to validate our Quantitative Basal Temperature© method—initially in a blinded fashion versus the serum LH peak (https://pubmed.ncbi.nlm.nih.gov/2364587/). As urine metabolites of estradiol and progesterone were able to be analyzed, we blindly validated the QBT© versus that. (https://pubmed.ncbi.nlm.nih.gov/19552997/).

We have repeatedly applied for funding to validate QBT© versus the ultrasound dominant follicle collapse without so far achieving that.

Although certain factors are known to affect first morning temperature measurements including sleep and illness, participants were instructed to record such external factors alongside their daily measurements. As well, shift workers were not included in the study since circadian rhythm disruptions are known to impact basal body temperatures. Furthermore, we have shown that the QBT© method is robust to massive variations in waking times in university students (https://pubmed.ncbi.nlm.nih.gov/19552997/).

D. Include definitions of terms and state reasons why two consecutive cycles were chosen instead of the standard (three). Include appropriate references.

To our knowledge, and certainly at the start of the original study in the mid-1980s, three was not the "standard" for number of cycles to document ovulatory status. We would be interested in the reference to which you refer, that three cycles of ovulation, are what is now accepted.

F. Include the rational for use of age group 20-41 years.

Irregular menstrual cycle timing, variable cycle length, and oligo-anovulation have been found to persist for up to 5-7 years after the onset of menarche in the setting of maturation of the hypothalamic pituitary axis over these years (Pena et al., 2018). Menopausal transition years are also marked by variable cycle lengths and increased prevalence of anovulation (Harlow et al., 2012). Although the women included in our study were proven to be normally cycling (QBT© data over two consecutive cycles prior to enrolment) the age range chosen allows for a wide range of menstrual cycle experiences to be evaluated while including women with cycles that have largely stabilized since puberty, but are likely several years outside of the menopausal transition period. (It may be of interest to you that the oldest person in this study did not become menopausal until she was 57).

Peña AS, Doherty DA, Atkinson HC, Hickey M, Norman RJ, Hart R. The majority of irregular menstrual cycles in adolescence are ovulatory: results of a prospective study. Archives of Disease in Childhood. 2018;103(3):235-9.

Harlow SD, Gass M, Hall JE, Lobo R, Maki P, Rebar RW, et al. Executive summary of the Stages of Reproductive Aging Workshop + 10: addressing the unfinished agenda of staging reproductive aging. Menopause. 2012;19(4):387-95.

REFERENCES

Thank you for these suggested minor referencing changes which we have made.

A. Line 358-359 : Remove the abbreviation of the journal , since you wrote the full name.

(See Line 393)

B. References 10, 11 and 17- complete the journal name

(See Lines 388 and 412)

GENERAL SUMMARY

Confirmation of ovulation occurs post ovulation which is controversial.

1. **The statement made on line 52, 53 and 54 can only be hypothesized by an autologous study with specific criteria met thereafter, using e.g the Bradford hill's criteria.** ***A retrospective cohort study cannot determine temporality.***

   This is secondary analysis of prospectively collected data. Thus, we **can** assess temporal changes across the documented phases of the 694 menstrual cycles we have analyzed.

   Lines 52-54: "Both women and clinicians need to know what is expected or 'normal' for menstrual cycle-related breast swelling and tenderness. We also need to know if breast changes across a cycle reflect ovulatory status"

   Please refer to response (1.) As our study followed women over the course of a year, with data collected prior to follow up/study outcome evaluation, it is by definition a prospective cohort design. Using daily self-reports from the MCD© describing menstrual cycle breast experiences with tracked menstruation dates and QBT© data proving ovulatory status, we were able to map out and characterize breast tenderness and swelling temporal changes across all cycles.

2. **The statement made on line 53, is not justified by your study design. To know if breast changes across cycle reflect ovulation status a comparative analytic study should be done.**

We have collected ovulatory status and we have collected breast tenderness and size changes. We can thus compare these two variables to make associations (not causes) between the two sets of variables.

3. **Write a sentence or two: how good physical health and emotional health were quantified.**

The original study enrolled women who were of normal body mass index (weight for height, 18.5-24.9), who were non-smokers, most of whom had jobs (at home or outside of the home) and who all reported that they were not ill with any chronic condition. Also, about 2/3 were doing more than average physical activity in the form of regular running exercise. An ill person is not able to do that exercise. We excluded compulsive exercise and inappropriate dietary patterns as our screening for emotional well-being. There are also associations of cycle lengths that are too long and ovulatory disturbances with illnesses, thus normal cycle lengths and ovulation are also, less specifically, admittedly, indicators of good health.

4. **What was your sampling technique? Probability or non- probability (consecutive sampling). These impacts the generalizability of your findings and nuances the conclusion.**

Please refer to the original study (ref 16). This was a community, convenience sample.

5. **The multiple linear regression analysis should not be used for categorical variables. For outcome that is categorical so a multivariate logistic regression model should be utilized.**

You are quite right. Thanks for catching this. The misunderstanding may have arisen from the wording in the Statistical Analysis section, which originally suggested a median split. We have now clarified this to accurately reflect that mean breast tenderness intensity is a continuous variable.

We respectfully clarify that for regression analysis we have used mean breast tenderness intensity which is a continuous variable, does not involve a median split of the data. Since the dependent variable is continuous, multiple linear regression is the appropriate statistical method to examine its relationship with the predictor variables.

We appreciate this careful review and have revised the Statistical Analysis section (Line 127) to ensure clarity.

6. **State the rational for combining two statistical analytic methods for this study. SPSS or R version can carry out the analysis as a stand alone.**

   I'm afraid this feels like this reviewer, despite many good suggestions, is becoming inappropriately picky. Our response: For some analyses SPSS is more convenient, and R is for others.

7. **Use standard tables and label them appropriately. Write important notes below the table.**

   Thank you for that suggestion.

   We have followed PLOS ONE guidance on the creation of Tables.

   Thanks to your good question, we have added the statistical test as a footnote to **Table 3**.

We appreciate the response to respond to requests by the editor on behalf of PLOS ONE, and to the two reviewers.

Sincerely,

(*on behalf of all authors)*

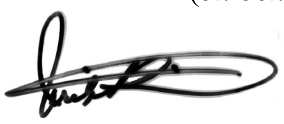

Jerilynn C. Prior BA, MD, FRCPC
Professor of Endocrinology and Metabolism
